# Complete genome sequence of European bat lyssavirus 2 detected in a Daubenton’s bat in Finland

**DOI:** 10.1128/mra.01034-25

**Published:** 2026-01-30

**Authors:** Ari Kauppinen, Tuija Gadd, Tiina Nokireki

**Affiliations:** 1 Animal Health Diagnostic Unit, Finnish Food Authority 52923, Helsinki, Finland; Katholieke Universiteit Leuven, Leuven, Belgium

**Keywords:** European bat lyssavirus 2, whole-genome sequencing, Daubenton's bat, rabies, phylogenetic analysis

## Abstract

We report the complete genome sequence of European bat lyssavirus 2 (EBLV-2) detected in a Daubenton’s bat in Finland in 2016. Analysis revealed a high nucleotide sequence similarity between the virus and previously reported Finnish EBLV-2 isolates from 1985 and 2009.

## ANNOUNCEMENT

European bat lyssavirus 2 (EBLV-2) has sporadically been detected in Daubenton’s bats (*Myotis daubentonii*) and a Pond’s bat (*Myotis dasycneme*) in the Netherlands (1), Switzerland (2), the United Kingdom (3), Finland (4), Germany (5), and Norway (6). EBLV-2 has caused two human cases: in Finland in 1985 (7) and in the United Kingdom in 2002 (8). The sequence reported here provides a valuable addition to the limited number of complete EBLV-2 genomes available.

EBLV-2 belongs to the genus *Lyssavirus* of the family *Rhabdoviridae*. Lyssavirus genome consists of a negative-sense single-stranded RNA, approximately 11.9–12.3 kb in length, and encodes five structural proteins (N, P, M, G, and L) (9).

EBLV-2 was detected in a female Daubenton’s bat found in the municipality of Inkoo, Finland, in 2016 using real-time RT-PCR (10). The bat exhibited severe ataxia and tetraparesis and was found grounded and dead later that day.

For virus RNA extraction, a spinal cord tissue sample was prepared as previously described (11, 12). RNA was extracted using the QIAamp Viral RNA Mini Kit (Qiagen) according to the manufacturer’s instructions without poly A carrier RNA. Extracted RNA was treated with the TURBO DNA-free Kit (Thermo Fisher Scientific). First-strand cDNA synthesis was performed using the SuperScript IV First-Strand Synthesis System Kit (Thermo Fisher Scientific) with random primers, followed by second-strand synthesis using the Second Strand cDNA Synthesis Kit (Thermo Fisher Scientific), according to the manufacturer’s instructions. The product was purified using the GenElute PCR Clean-Up Kit (Sigma-Aldrich) and quantified using Qubit (Thermo Fisher Scientific).

A sequencing library was prepared using the Nextera XT DNA Library Preparation Kit (Illumina) as previously described (11, 12) and purified with AMPure XP Beads (Beckman Coulter). The library was quantified using Qubit and the NEBNext Library Quant Kit for Illumina (NEB), and the average fragment length was determined using the Agilent High Sensitivity Bioanalyzer Kit. The indexed library was paired-end sequenced on an Illumina MiSeq platform using the MiSeq Reagent Kit v3 (600 cycles, Illumina).

The sequence data were analyzed using CLC Genomics Workbench v25.0 (Qiagen) with default parameters. Briefly, raw reads were quality trimmed, and adapter sequences were removed. Host-derived reads were filtered out by mapping to Daubenton’s bat genome (GCF_963259705.1), and the remaining trimmed reads were mapped against a reference sequence (KY688151). Within CLC, the Local Realignment tool was used to realign reads around indels, and the outputs were used to extract consensus sequences. Predicted proteins were annotated using the Annotate with BLAST tool. The genome termini were validated by manual inspection and showed no mixed bases, gaps, or unique mismatches relative to the published EBLV-2 reference genomes. A comparison of the genomes with previously published viruses was conducted using the online Basic Local Alignment Search Tool nucleotide (BLASTn) against an NCBI nucleotide database (June 2025), applying default settings.

**Fig 1:**
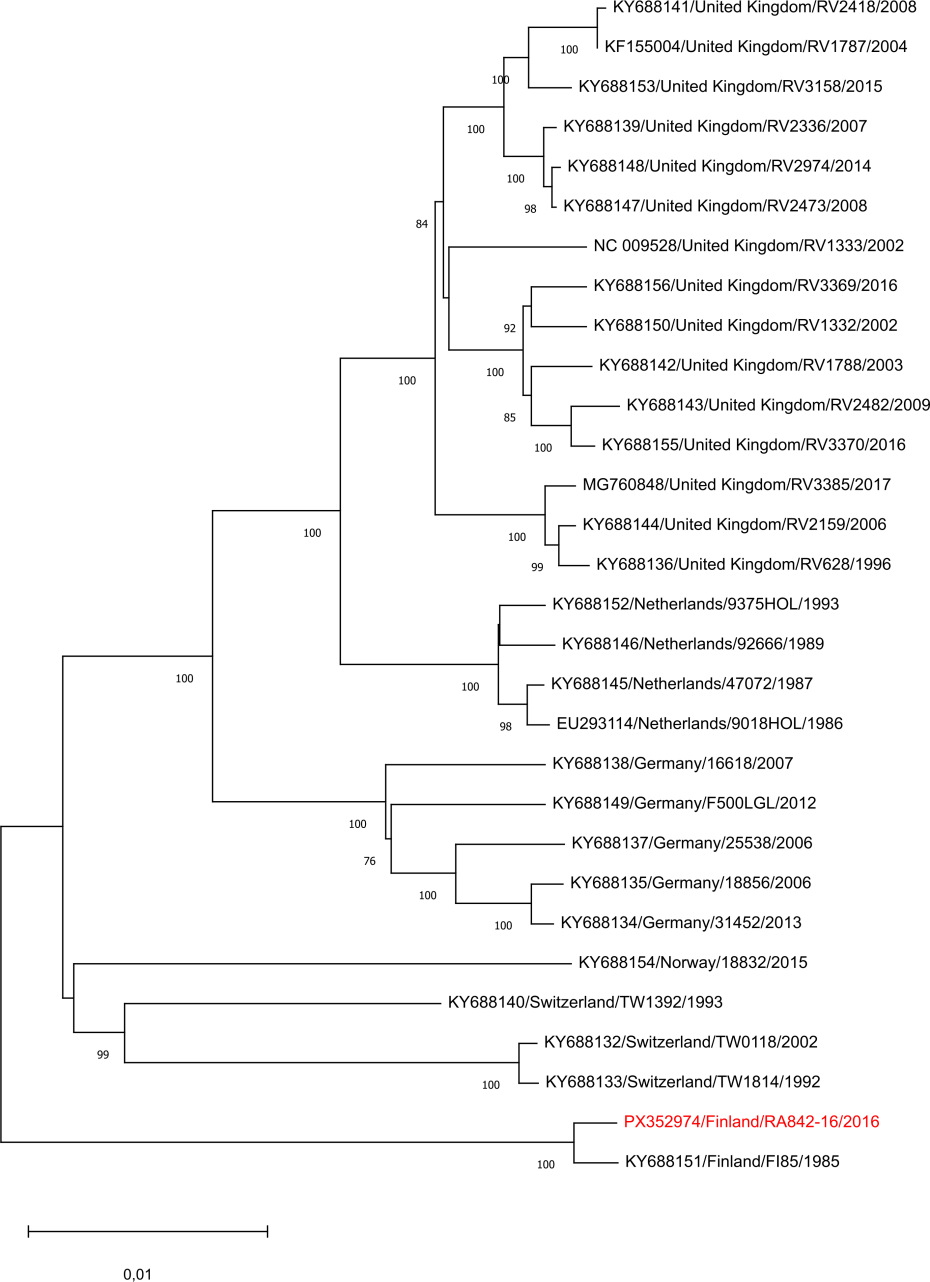
Phylogenetic tree of the complete genomes of representative EBLV-2 isolates. The GenBank accession number, the country of origin, and the year of isolation are given for previously published EBLV-2 sequences; the sequence reported in this study is indicated in red. Alignments of the complete genomes were produced using the MAFFT program v7 (13) with default parameters, and the phylogenetic tree of the aligned sequences was constructed in MEGA v11 by applying the maximum likelihood method (14). The numbers at the nodes of the tree indicate bootstrap values of 1,000 replicates; values under 70 are not shown. The scale bar indicates nucleotide substitutions per site.

Table 1 and Fig. 1 summarize the assembly characteristics and illustrate the phylogeny of the EBLV-2 genomes. The analysis revealed high nucleotide identity (99.63% and 99.69%) with the previously reported Finnish EBLV-2 isolates KY688151 and JX129232, respectively.

**TABLE 1:** EBLV-2 genome assembly parameters and their values

	RA842-16
Sample material	Spinal cord
Date of collection	5 October 2016
Ct value (real time RT-PCR)	21.34
Sequencing platform	Illumina MiSeq
Reads	Paired
Average read length (trimmed)	219
Average sequencing depth	7,074×
Read number (total)	3,260,286
Read number (mapped)	398,755
GC (%)	44.6
Assembly length	11,927
Completeness (%)	100

## Data Availability

The EBLV-2 genome sequence was deposited in the NCBI GenBank with the accession number PX352974. Corresponding raw reads are available under the accession number PRJNA1327112 in the SRA database.
